# Researches on the Paralysis Attendant on Insanity

**Published:** 1827-04

**Authors:** 


*De la Paralysie, considerée Chez les Aliénés; recherches faites dans le Service de M. Royer-Collard et de M. Esquirol.* Par F. L. CALMEIL, Docteur en Medecine, &c. Premier Interne, en Medecine, à la Maison Royale des Alienés de Charenton. 8vo. pp. 446. Paris, 1826, and Balliere, Bedford-street, Bedford-square, London.

*Researches respecting the Paralysis attendant on Insanity, made under the eye of Royer Collard and Esquirol, in the Charenton Lunatic Asylum.* By Dr. CALMEIL, of that Establishment.

THE receptacles of the insane present a great number of their unfortunate inhabitants deprived, more or less, of their muscular power, as well as of their intellectual faculties. In some, the speech is embarrassed—others walk in a staggering manner, or require a stick—some are hemiplegic or paraplegic—others have little power of any of their limbs—and some are observed with contractions of one or more members. All these disorders of locomotion have been alluded to by writers on insanity under the term *paralysis*. But Dr. Calmeil thinks that we have thus confounded together things that are different. Modern investigations have proved that almost all severe paralytic affections are dependent on organic lesions of the cerebro-spinal system, as hæmorrhage, softening, abscess, sanguineous or serous congestions, wounds, tumours, hydatids, tubercles, or osseous encroachments. It is the object, therefore, of our author, to investigate the conditions of the brain and spinal marrow on which the paralyses of the insane depend. Dr. C. appears to be careful in making his observations, and cautious in drawing his conclusions. He admits the difficulty of obtaining accurate or authentic histories of the insane, and therefore suspends his judgment on points where he is not quite certain of the data.

The following inferences, however, he thinks he may draw with perfect safety, as founded on the broad basis of experience.

1mo. Paralyses are very frequent in the insane.

2ndo. The organic lesions which occasion them are almost always situated in the brain itself.

3tio. These lesions are generally as follow: chronic inflammation of the cerebrum, especially on its surface, and in its envelopes—cerebral or meningeal hæmorrhages—sanguineous congestions—softenings of the cerebral and spinal masses—encysted abscesses.

There is no doubt that tuberculous and hydatid growths, &c. may be found in the brains of the insane, as well as in other people; but such appearances are rare, and cause but very few deaths.

By the term "general paralysis of the insane," our author means, in fact, chronic inflammation of the cerebral pulp, but he prefers the term "general paralysis," as expressing a disorder of function, or an effect which will admit of no litigation in itself, though people may entertain different opinions respecting the nature of the organic lesion which produces it. In this, we think Dr. Calmeil has acted wisely.

This General Paralysis of the Insane, is divided by our author into three degrees of intensity, and, in illustration of these degrees, he details between seventy and eighty cases, so that the reader may see the facts on which he grounds his division. These cases form no bad corpus historicum of insanity itself—for whether we regard this disease as a complication or cause of the mental aberration, the facts are not the less valuable, since both the intellectual and corporeal phenomena are detailed. We cannot, however, in this analysis, go into an account of these cases, as they occupy three fourths of the volume; but may, if our space will admit, give a few samples at the end of this article.

1. *General Paralysis in its first degree, or most simple form.*

Embarrassment in the movements of the tongue is sometimes the first symptom of the general paralysis which is to succeed, and is often well marked before any other symptom of paralysis in the limbs becomes apparent. The speech is not clearly articulated—the patient is obliged to use some efforts in getting out his words, as if he was intoxicated. If asked to put out his tongue, there is little or no deviation to any side perceptible. The mouth is not drawn from its place, nor are the features in the least distorted—in fine, there is only an embarrassment of the speech.

*Second degree of Intensity.*

The symptom above described having continued a certain time, the motility of the tongue becomes more restrained, and we are obliged to divine, as it were, what the patient is saying, or trying to say. He cannot distinctly pronounce any word, and one may plainly see that the tongue is paralyzed. If the patient be sitting, and we desire him to walk across the room, he applies his hands to the sides of the chair or sofa, and raises himself very slowly. When up, he does not step out with confidence, but hesitates, like a child who is just beginning to walk. He goes forward, but sallying from side to side, with unsteady gait. The lower extremities are more affected, in proportion, than the upper; the head droops forward, and the body does not rest in equilibrium on the pelvis. These individuals see, feel, and perceive, but the energy of the senses is much impaired. The skin may be strongly pinched before they express any pain—odours must be powerful before they affect the olfactories, and the patient is often incapable of distinguishing unpleasant from agreeable smells. It is evident, in short, that the portions of brain which preside over sensation are equally affected as those which preside over locomotion.

People, in this second degree of paralysis are generally in a state of *dementia*. Their *malpropreté* is extreme, and they are constantly soiling their clothes with their own evacuations. This does not appear to be always dependent on paralysis of the sphincters of the bladder or rectum; for our author has known instances where the urine has come away guttatim, while the greatest difficulty was experienced in overcoming the sphincter ani, either by purgatives or enemata.

During the course of this as of the former period, the general health appears but little affected. The tongue is moist, the appetite considerable, the digestion easy, the heat of skin natural, the pulse moderate and regular—in short, the disease of the brain has yet caused little or no disturbance in the functions of the body, with the exception of those pointed out.

### *Third Degree of Intensity.*

Nothing can be more deplorable than an insane person in the third degree of general paralysis. Those lesions of the brain which disturb the intellect as well as the locomotive powers, have now reached their highest grade. The wretched patients are incapable of motion, and prostrated both in mind and body to a state of negative life—or rather a species of slow agony (espèce d'agonie lente) during which the body dies piecemeal, presenting the most horrible spectacle of human misery and imbecility!

In some people the tongue is so paralyzed that they cannot utter a single word, and only send forth vague and confused sounds. The lower extremities refuse all support to the body—and in ulterior periods of the complaint, they cannot even move the limbs in bed. The upper extremities are not paralyzed to the same extent. In these conditions, there is often scarcely a ray of intelligence left, and the attendants are obliged to feed the patients by force. The excrements are passed involuntarily and unnoticed—no impression is made on the individual by external objects, though he still has some slight power of hearing, seeing, tasting, and smelling. Of this deplorable stage of the disease there are, of course, many varieties in degree, according to the progress which the encephalic malady has made.

In respect to the interior functions of the body, the following observations are recorded by our author. The digestion, which was so active in the beginning of the malady, now languishes, the strength diminishes, the flesh wastes, eschars form on the back, &c. dropsical swellings take place in the scrotum, lower extremities, and abdomen—and, finally, some of the organs of the chest or abdomen give way, the lungs or the gastric and intestinal mucous membrane, and then death soon terminates the scene.

Let us now proceed to examine into the alterations found in the brains of those who die in this state of general paralysis.

1. *Vascularity of the Cranium.* Sometimes, on stripping the dura mater from the internal surface of the skull, the blood will ooze out in small drops; and the same is occasionally seen when the pericranium is detached. The spongy texture, or centre of the bone is usually found red. On the other hand, the plates of the skull are sometimes much attenuated, or almost perforated, especially about the vertex, occasioned, in general, by the growth of vegetations from within.

2. *Injection, &c. of the Dura Mater.* When this is separated from the skull, it is sometimes found covered with blood. If this be lightly sponged off, the vessels of the dura mater will be found very much injected, and the sinuses distended with black blood. Sometimes it is perforated, the same as the cranium, by the growth of vegetations.

3. *Vegetation of the Pia Mater.* Granulations of this membrane seldom attract much attention, yet they sometimes become so developed as to be well worthy of consideration. They have been seen as large as the end of one's thumb, and are usually found about the edges of the fissura magna sylvii.

4. *Serous Effusion into the Cavity of the Arachnoid.* This is an almost invariable occurrence where there has been general paralysis. The effusion occupies chiefly the basis cranii, but is more or less diffused over all the lobes of the brain, wherever the arachnoid extends. The serosity is sometimes turbid, or even inclining to be sanguinolent, but is generally limpid.

5. *Albuminous Concretions* are occasionally found developed in the arachnoid, in the form of a consistent jelly, without any trace of organization. They are of various consistency.

6. *Organized False Membranes.* The albuminous concretions formed between the sheets of the arachnoid are organizable. After being a certain time in contact with the serous membrane, these concretions become vascular, and adherent to the arachnoid reflected over the pia mater. These false membranes are sometimes red, sometimes pale. They vary in thickness, and are generally found on the sides of the hemispheres—rarely towards the base of the brain.

7. *Encysted Concretions, with Hæmorrhage.* This species of morbid alteration has been often met with by our author. It consists of a coagulum of blood coated by a layer of coagulable lymph resembling a cyst.

8. *Sanguineous Infiltration between the Laminæ of the Arachnoid.* Our author found only one example of this in his numerous dissections. The infiltration was only over one hemisphere.

9. *Œdema of the Arachnoid.* This is hardly ever absent. When the dura mater is removed, we see, in the intergyral spaces, a transparent liquid, raising the arachnoid from the pia mater beneath. It appears to be jellied, and trembles when shaken, but oozes out when the membrane is incised.

10. *Thickening of the Pia Mater and Arachnoid.* In a sound brain, the arachnoid lies so close to the pia mater, and is of such a delicate structure, that it is almost always torn when we attempt to raise it with the point of a probe or scalpel. In the insane affected with general paralysis, this membrane often acquires a considerable degree of thickness and tenacity —sometimes giving it an appearance not very unlike that of the dura mater.

11. *Injection of the Arachnoid and Pia Mater.* This phenomenon is frequently observed. The trunks of vessels seem gorged—the capillaries injected—the membranes themselves of a reddish colour, which they preserve when held up to the light, or even when they are dried.

12. *Adhesion of the Cortical Substance to the Pia Mater.* This is extremely common, and is the most extensive alteration in the kind of disease under consideration. It is almost invariably found, when we dissect carefully, around the whole brain. Sometimes, however, it is only partial, or more firm in one part than in another. When we attempt to pull up the pia mater, it refuses to be raised, or drags a portion of brain with it.

13. *Consistence of the Cortical Substance.* This is almost always less than in health, and is sometimes reduced to the consistence of a rotten apple. This softening generally extends to the depth of a quarter or half a line into the cineritious substance. Sometimes it is very superficial, being little thicker than a coat of varnish.

14. *Colour of the Cortical Part.* This is very various, but rarely pale. It generally presents an erysipelatous, rosy, lilac, violet, or deep red hue. It is also strongly injected.

15. *Consistence and Colour of the Medullary Matter.* This was most frequently found by our author to be of the usual healthy consistence. In some cases, however, he has found a certain degree of density in the corpus callosum. The same may be said of the colour of the medullary mass. In the majority of cases it is not altered from the natural appearance.

16. *Local Lesions of the Brain.* These are rare, only three examples presented themselves among our author's dissections. One was the cicatrix of an old extravasation of blood—another shewed an erosion or ulceration on the surface of the brain—and the third a tubercle.

17. *Serous Effusion in the Ventricles.* This is a very common occurrence, varying in quantity from one to several ounces. It is generally in proportion to the serous effusion between the meninges. The lining membrane of the ventricles is often very red, and covered with villosities.

18. *The Spinal Canal.* There is generally serous effusion here as well as in the head; but the substance of the medulla oblongata and spinal cord is usually found unaltered.

The above morbid appearances, although they do not satisfactorily account for the symptoms of general paralysis, inasmuch as they are not uniform nor constant, yet, our author thinks, with reason, that they are sufficient evidence of the pre-existence of chronic inflammation in the brain and its envelopes, especially in the cortical substance, and that this chronic phlegmasia is the cause of the general paralysis, by producing organic lesions, of one kind or other, in the encephalon. But granting that no appearance of inflammation is observable in the brain, on dissection, he thinks it is no proof that it did not formerly exist there. The inflammation itself may disappear, yet leave the organic changes behind which produce the general paralysis.

Dissection, he observes, will always furnish arguments for the advocates of "nervous paralysis," or the lesion of a function without lesion of the structure on which that function depends. There can be no question that the disturbance of a *function* must depend on its *organ;* but the alteration in the organ is not always discoverable by the eye, and, as we have said, on a former occasion, it is still convenient to have the distinction between structural and functional lesions retained. Would it not be absurd to say that a man's stomach is *altered in structure,* because a piece of bad news annihilates his appetite in the middle of his repast?

### *Aspect of the Mental Alienation in the Course of the general Paralysis.*

Nothing is more difficult than to classify or arrange the phenomena of mental alienation, so much do they vary in different individuals, and at different periods in the same individual.

1. *Dementia, or Weakness of Intellect.* Almost all those affected with general paralysis, present, from the beginning, a more or less marked state of debility in the intellectual functions. The memory is treacherous, there is an inaptitude for the usual occupations of mind and body—they attach little importance to affairs the most serious, and are unable to comprehend a discourse that is in the least degree abstruse. Their own narratives are full of diffuseness and incoherency. Sometimes the patient is calm and tranquil from the beginning to the end of the disease; but this is not frequently the case. In the greater number of instances, there is—

2. *Dementia and Monomania joined.* The domination of an *exclusive idea*, if we may so term it, is very remarkable in the majority of cases of general paralysis, and often shews itself sometimes before this last symptom appears. These individuals are filled with the idea of grandeur and riches, which they suppose themselves to possess. Intoxicated with their own good fortune, they give themselves up to the most romantic visions and illusions, and their inward joy is manifested in every action, more especially as they believe that all around them partake in their felicity. They are prodigal of places, pensions, riches, and honours to all their friends—in short, they are infinitely more happy than those who actually possess all these good things, but who are cursed with reason and reflexion as accompaniments! These monomaniac ideas of riches and grandeur seldom continue to the third period, in general paralysis. At this epoch there is a complete abolition of intellect, and the happy dream vanishes for ever. In a small proportion of cases the monomaniac hallucination is of the sombre cast, of which some curious examples are given by our author.

*Dementia and Mania.* In a certain proportion of cases, there is violent mania joined with dementia. The agitation and fury of some individuals are extremely difficult to govern.

This state sometimes commences with the symptoms of general paralysis, but more frequently supervenes on it.

*Duration and Progress of general Paralysis.*

This is various, depending, of course, on the slow or rapid march of the cerebral lesion. Some paralytics live eight months, a year, 18 months—some will resist the malady two or three years, but seldom longer than this. The medium duration of life, in this disease, was thirteen months, according to our author's observations. When an individual, however, becomes affected with this terrible complaint, his life is very precarious. To-day he can walk, the debility of the lower extremities appears inconsiderable, and his language can be understood—to-morrow he will be quite incapable of walking, or of articulating a single word. At a time when one would prognosticate that he would live a year, he will be within a few days of his end. The reverse of this is sometimes the case. A man will be rapidly hastening to his grave, in all appearance, and yet the disease will take a sudden turn for the better—but this is rarely of long standing; a relapse is the general result.

*Of Prognosis and Termination.*

We shall pass over the diagnostic marks of this disease, since they are sufficiently obvious in the description of the malady. In respect to the prognosis, it will readily be perceived that little hopes can be entertained of recovery from this terrible visitation. Royer-Collard, the Elder, who had so much experience of insanity, was in the habit of pronouncing at once the total incurability of general paralysis among the insane. In the course of 20 years' observation he never knew an instance of recovery, under such circumstances. Esquirol is of the same opinion. The observations of our author tend to confirm this melancholy prognosis.

*Predisposing Causes of General Paralysis.*

The influence of sex on this complaint is very great. We see the disease certainly among the female insane; but among male lunatics we are surrounded, as it were, with the malady. Out of a mass of twelve hundred men, eighty cases of general paralysis were found—or one in fifteen. In five hundred female lunatics, there were only discovered ten cases of the disease—or one in fifty. It is very difficult to account for this disparity. Our author has not observed the disease under consideration, in any patient under the age of 32 years. He has seen fourteen examples of it between the age of 32 and 40—seventy examples between forty and fifty—nine between 50 and 60.

### *Exciting Causes.*

Of all professions, that of arms has had the most marked and fatal influence, as an exciting cause of this malady. A great number of Napoleon's troops have died of this disease; nor did it respect any rank or class of the military! Our author attributes this disposition on the part of the soldiery to the fatigues of war—the excesses into which this class are often plunged—the moral vivid emotions consequent on mortal combats. The Douaniers (Custom-house Officers) are very subject to it, probably from their irregular modes of life, in watching at night along the shores, exposed to the nocturnal skies. Out of five Douaniers who came into the Charenton last year, four were affected with general paralysis, and the fifth shewed symptoms of its approach. Exposure to heat, as in the employment of founders, cooks, bakers, glass-makers, &c. has a marked influence in the production of this disease. Moral causes, also, as the passions, in excess, are peculiarly powerful in exciting the disease. So are excesses in wine, women, spirituous liquors, &c.

### Treatment.

It may seem almost unnecessary to take up the subject of treatment in a disease which is considered uniformly fatal, in a shorter or longer period of time. Our author thinks, however, that we should not abandon these cases to nature; and that it is probable that, if recent cases were properly treated, success might sometimes crown our efforts. In this we agree with our judicious author. It is not much to be wondered at, indeed, that these cases should seldom or ever be cured by the usual plans which are pursued. The symptoms of muscular debility, so conspicuous in these cases, have led to wild, and, perhaps, dangerous medications of an excitant or stimulating nature, under the vain hope that we may give tone to paralyzed muscles, when the source of nervous influence is in a state of disease:—Witness the attempts made to excite paralyzed parts by electricity, nux vomica, strichnine, &c.

Viewing the proximate cause of the disease in the brain as a chronic inflammation of that organ or its coverings, our author advises the abstraction of all stimulation. For this purpose, the patient should, of course, be separated from his friends and connexions, where he is not only improperly fed, but where the passions are constantly excited, by circumstances which ought to be kept entirely out of his view. The lightest kinds of food—the most diluent and mild species of drink—gentle cooling aperients—warm semicupia—cold applications to the head—local, or even general bloodletting, especially if the patient be of a full habit of body—blisters and other modes of counter-irritation at some distance from the head—setons or other permanent drains in the nucha—the moxa or actual cautery:—These and analogous measures ought to be tried in the early periods of general paralysis, especially where it has come on coeval with, or not long after, the disorder of the intellectual functions.'

We shall now introduce to our readers an example or two from each of the three degrees of intensity into which our author has divided this disease.

*Case* 1. Le Sieur Jean Baptiste P———, a gens d'arme, aged 46 years, of tall stature, and moderately strong constitution, entered the Military Hospital, complaining of cephalalgia, ringings in the ears, and evincing incomplete paralysis of the tongue, with some embarrassment in walking. On examination, it was found that he had some fixed erroneous ideas—that he slept very little at night—and that he talked much to himself by day. A blister was applied to the nucha, and leeches were applied to the temples, but without relief. As the heat of summer advanced, the disorder increased, and he was sent to the Asylum. He was there questioned as to his history; but no satisfactory answers could be procured. His memory and judgment appeared abolished. He now lapsed into a state of dementia, unaccompanied by any violence. He could not walk with any degree of security—yet the senses of sight, hearing, smelling, and touch, although diminished in energy, still existed. All the other functions of the body seemed little affected. The intellect and the power of loco-motion alone were impaired. This is a specimen of the disease in its first or mildest degree.

*Case* 2. This and the foregoing case were both under M. Esquirol.

B———, a merchant, aged 47 years, was addicted to drink in excess, and he was of a very violent and irascible temper. He was in possession of sufficient wealth, and could have no just dread of reverses. He had previously enjoyed good health, and had no hereditary disposition to insanity. In 1821, and without any apparent cause, he became a monomaniac. He thought of nothing but murders, conspiracies, and poisonings. He misconstrued every thing he saw or heard, and was constantly imploring the interference of the magistrates—writing to the minister of police, &c. These were considered by his friends as eccentricities, and no medicinal or other measures being taken, the disease passed into a chronic state. In 1823, the malady took quite a different turn. The man believed himself possessed of immense riches—offering gold and jewels to his friends in great abundance. He also conceived himself to be a genius of the first rate. For him was reserved the honour of accomplishing all those enterprizes in which the ablest men had failed. He began by rebuilding the Tower of Babel; and soon became so troublesome to society, that he was put under restraint, and sent to the Charenton Asylum. Here he appeared very tranquil, and was busily employed in drawing plans of the Tower of Babel. But he soon evinced a considerable difficulty in pronouncing his words, and an inability of walking with a steady gait. The lower extremities gradually lost power, and he could only walk with the aid of a stick, or leaning on tables, chairs, &c. Still he was sensible to pain, he had the power of hearing, seeing, &c. and the various functions of respiration, circulation, digestion, secretion, &c. went on well. During the year which he has passed at Charenton, he continues precisely in the same state.

These two are sufficient samples of the first degree of intensity, in general paralysis of the insane.

### *Second Degree.*

*Case* 3. A——, a lieutenant of the line, aged 42 years, had made fifteen campaigns under Napoleon and his Generals, always with the character of being a most excellent officer. Of late years, he had experienced severe moral afflictions, having been deprivent of his rank and honours by circumstances totally independent of himself. These made a deep impression on his mind, and all the efforts of philosophy were exerted in vain to bring peace to a wounded spirit. During eight months he was treated in a provincial hospital; but the symptoms increasing in force, he was sent to the Charenton Asylum. The servant who attended to him all the time he was in hospital, informed M. Royer-Collard, that he had difficulty in pronouncing words from the time he first went there, and that he frequently passed his fæces and urine in bed or in his clothes, apparently unconscious of the circumstances. He also stumbled when any obstacle touched his feet in walking.

In the Charenton, he came up to our author with a staggering gait, and demanded his discharge from the Asylum, in a faultering accent, in which he could hardly pronounce more than one syllable in each word —in short, the paralysis of the tongue was quite evident. When upright, he could walk tolerably well; but in sitting down or getting up he staggered, and nearly fell at each attempt. He moved his limbs about in bed, but the muscular debility was unequivocal. He had the use of the five senses: but his intellectual functions were greatly deteriorated. His memory, judgment, and affective passions all disappeared; and, whether from inattention, or want of power in the sphincter muscles, he frequently soiled the bed and his clothes by his excrements and urine. He had fits of great agitation. Night and day he sent forth lamentable exclamations, uttered without any coherence of ideas. His actions were also very extravagant sometimes. All the vital and natural functions were carried on with regularity. At present, it is impossible to understand a word he says—the body is inclined to one side—and he frequently falls down in his attempts to walk.

### *Third Degree.*

*Case* 4. Victoire L——, aged 56 years, had ceased to menstruate when in her 36th year. She has borne children, and had no hereditary predisposition to insanity. After many years of melancholy attendance on her husband, who died of phthisis, she lost her health, and was the subject of severe vicissitudes of fortune. For twenty years past she has been subject to periodical nervous attacks, sometimes ending in seizures of a convulsive character, but without loss of sense. At the age of 53, her memory began to fail, but still she attended to her domestic affairs, and shewed no symptoms of insanity. At 55, mental derangement became unequivocal. She talked to herself—uttered all kinds of nonsense, and did not know her children. She was then confined. Our author examined this woman four months after the insanity had commenced. She could not pronounce the final syllables of words. The tongue was paralyzed. Dr. C.'s attention was then directed to the lower extremities, and found that the patient could not walk without leaning against the wall, or on some chair or table in her way. The sensibility of the skin, the power of digestion and that of the other functions were still preserved. The mental alienation and the defect of loco-motive power were the prominent characters of the complaint. The patient could not tell her name or her age—she had no consecutive train of ideas. Yet she was tranquil. In the mornings she was dressed, and then assisted to a sofa, where she continued till she was taken to bed. She would eat when victuals were presented to her, but never asked for them. She had not the least regard to cleanliness, but passed her excrements in bed or on the sofa, apparently without the least consciousness. She was obliged to be treated exactly as if an infant.

Six months rolled away, and still her physical health was tolerably good; but the cerebral disease made progress. The rectum would not now propel its contents, and the urine was constantly dribbling away. She was utterly incapable of walking, and even the upper extremities had little power of motion. Her existence was purely automatic. Eight months from the time she entered the Charenton, and eighteen months after the commencement of the mental alienation, this wretched piece of vegeto-animal mechanism ceased to exhibit symptoms of vitality.

*Dissection.* The cavity of the arachnoid contained much serosity, calculated at four ounces. There were vegetations on the surface of the brain, which traversed the dura mater, and had worn impressions on the inner surface of the skull, as far as the diploë. Some of these vegetations were as large as the end of the thumb, with a ragged unequal surface. The vessels of the brain were not at all injected. The pia mater separated without any difficulty from the brain, except near the optic nerves, where the attachment was inseparable. In some places, the cortical substance was of a dark red colour. The lateral ventricles contained two ounces of water. The 4th ventricle was covered with granulations. The cerebellum, tuber annulare, and medulla oblongata presented no alteration. The spinal marrow was examined; but the appearances were not satisfactory. The cineritious substance was strongly coloured, and resembled the lees of wine. There was no other morbid appearance in any part of the body.

We shall now conclude. We had considerable difficulty in drawing up this analysis, in consequence of the strange and confused arrangement of the author. But we consider the facts and observations as valuable on this particular point of pathology, the author having had very superior opportunities of investigating the phenomena and post-mortem appearances in the paralysis of the insane. This very striking and frequent accompaniment of mental derangement is far from being well understood by any except those who have the care of the insane. But it is very important for private practitioners, of every class, to be well aware of its nature and of its dangerous character. On this account we think we have not wasted our time or that of our readers, in condensing within a very moderate space the practical and valuable information contained in a volume of nearly 500 pages.

